# Clonal Astrocytic Response to Cortical Injury

**DOI:** 10.1371/journal.pone.0074039

**Published:** 2013-09-10

**Authors:** Eduardo Martín-López, Jorge García-Marques, Raúl Núñez-Llaves, Laura López-Mascaraque

**Affiliations:** Instituto Cajal, CSIC, Madrid, Spain; Hospital Nacional de Parapléjicos - SESCAM, Spain

## Abstract

Astrocytes are a heterogeneous population of glial cells with multifaceted roles in the central nervous system. Recently, the new method for the clonal analysis Star Track evidenced the link between astrocyte heterogeneity and lineage. Here, we tested the morphological response to mechanical injury of clonally related astrocytes using the Star Track approach, which labels each cell lineage with a specific code of colors. Histological and immunohistochemical analyses at 7 days post injury revealed a variety of morphological changes that were different among distinct clones. In many cases, cells of the same clone responded equally to the injury, suggesting the dependence on their genetic codification (intrinsic response). However, in other cases cells of the same clone responded differently to the injury, indicating their response to extrinsic factors. Thus, whereas some clones exhibited a strong morphological alteration or a high proliferative response to the injury, other clones located at similar distances to the lesion were apparently unresponsive. Concurrence of different clonal responses to the injury reveals the importance of the development determining the astrocyte features in response to brain injuries. These features should be considered to develop therapies that affect glial function.

## Introduction

Damage to the central nervous system (CNS) is classified in two general groups depending on the type of injury. Injuries involving rupture of the brain blood barrier (BBB) along with bleeding and edema are called anisomorphic or “open” lesions. These injuries produce structural damage of the CNS and group all traumatic injuries and strokes. On the other hand, lesions that do not disrupt the BBB, and usually do not involve bleeding, are called isomorphic or “closed” injuries [1]. Examples of these injuries are the multiple sclerosis or the toxic damage. Both types of injuries trigger a cascade of cellular and molecular events trying to restore the CNS homeostasis to minimize the tissue damage. In traumatic injuries the damaged tissue is rapidly enclosed by the generation of a *glial scar*, a barrier mainly constituted by reactive astrocytes, microglia, fibroblasts, pericytes, endothelial and meningeal cells [2]–[4]. Among these cells, astrocytes react by exhibiting hypertrophy of their cellular processes and hyperplasia (proliferation), a process referred as reactive astrogliosis [5]–[8]. Reactive astrocytes increase their cytoplasmic content overexpressing glial fibrillary acidic protein (GFAP), vimentin and S100β intermediate filaments besides inhibitory molecules such as chondroitin sulfate proteoglycans [9]–[12]. Reactive astrocytes undergo alterations in phenotype and gene expression depending on parameters such as distance to the injury, indicating a high degree of heterogeneity [13], [14].

The heterogeneity in astrocytes is not exclusive of reactive cells. The concept of astrocyte heterogeneity emerged in the late nineteenth century based on morphological differences [15]. During the last two decades, the development of new immunocytochemical and molecular techniques allowed identifying differences between astrocytes from the same or different CNS regions, which revealed the importance of the heterogeneity in astrocytes as an intrinsic feature at the morphological, molecular and functional levels [16]–[19]. This heterogeneity is also reported in reactive astrocytes located close to or surrounding an injury in the CNS [20]–[22]. Although knowledge about the biochemical profiles of reactive astrocytes is increasing, the origin of these cell populations is still under discussion. Some authors reported a limited cells division confined to the penumbra area [23], while others suggested that they arise from preexisting astrocytes [24].

Recently, the development of a novel tool for the analysis of astrocyte clones, the *Star Track* method, evidenced the relationship between heterogeneity and lineage in astrocytes [25], although it is unknown whether the cell lineage also determines the heterogeneity in reactive astrocytes. To address this issue, in this work we used the Star Track method to study the relationship between the phenotype and heterogeneity of reactive astrocytes with their ontogenetic origin [25]. Additionally, we performed an analysis of cell division to study the proliferative response of astrocyte clones to the injury.

## Materials and Methods

### Animals

C57 mice were obtained from the Cajal Institute animal facility. Animals were housed in standard cages, maintained under to 12 h controlled light-dark cycles with food and water available *ad libitum*. All procedures were in compliance with the guidelines for animal care of the European Union (2010/63/EU) and approved by the Bioethical Committee at the Spanish National Research Council (CSIC).

### In Utero Electroporation

**Figure 1:**
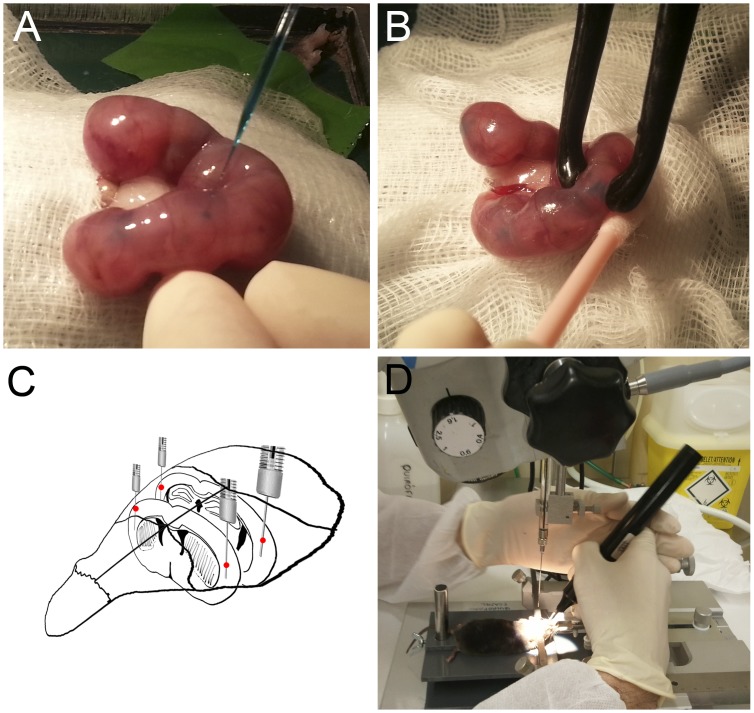
Procedure for *in utero* Star Track electroporations at E14 and cortical lesion in adult mice. **A–B.** A pregnant mouse was anesthetized at E14, and the uterus was exposed. **A.** The plasmid mix solution is *in utero* injected into the lateral ventricles of the cerebral cortex of each embryo. **B.** *in utero* electroporation. **C.** Brain injuries were practiced at 0 and −2 mm AP/3.5 mm ML stereotactic brain coordinates from bregma in 2–3 months adult mice. **D.** Lesions were performed after electroporation in both hemispheres using a stereotactic frame by inserting a microliter syringe attached to a 22-gauge needle and mice were allowed to survive for one week after surgery. **E.** Scheme of Star Track plasmid mixture injected at embryonic stages.

Pregnant mice at embryonic day 14 (E14) were deeply anesthetized by isofluorane inhalation (Isova® vet, Centauro, 2 ml/L) and maintained at 37°C. Uterine horns were exposed and the location of the embryos was visualized by trans-illumination through a optical fiber. Plasmid mixture (2 µl, 2–5 µg DNA/µl containing 0.1% fast green) was injected into lateral ventricles (LV) using a glass micropipette (Fig. 1A). Then, each embryo was hold between tweezer-type electrodes (Fig. 1B) to deliver one or two trains of 5 square pulses (35 V; 50 ms followed by 950 ms intervals). Uterine horns were placed back into abdominal cavity. Abdominal incision and skin were sutured with absorbable polyglicolyc acid (Surgicryl, Hünningen, BE) and silk (3/0 Lorca-Marin, Murcia, ES) sutures respectively. Skin was cleaned with povidone-iodine and pregnant mice received a subcutaneous injection of both 5 mg/kg of the antibiotic enrofloxacine (Baytril; Bayer, Kiel, DE) and 300 µg/kg of the anti-inflammatory/analgesic meloxicam (Metacam; Boehringer Ingelheim). Injected embryos were allowed to develop normally and were analyzed at selected adult ages.

### Fine Needle Cortical Injuries

Adult mice (2–3 months) were deeply anesthetized with equithesin (30 ml/kg) and their heads were placed in a stereotaxic frame. A skin midline incision was made on the skull, and lidocaine (Xilonibsa Aerosol, Barcelona, ES) was applied locally. Four skull holes were drilled bilaterally at the following stereotactic coordinates from bregma: 0 and −2 mm AP/3.5 mm ML (Fig. 1C, D). Lesions, consisting in cortical holes, were practiced by the insertion of a 22-gauge needle syringe (Hamilton 7101N) through the cranial holes at 2 mm deep for 2 min. (brain-injured mice; N = 20). Sham animals underwent similar procedure except the cortical injuries (sham-injured mice; N = 10). Afterwards, skin was sutured and postoperative cares included injections of enrofloxacine (5 mg/kg, Bayer, Kiel, DE) and buprenorphine (8 µg/kg, Buprex, Merck & Co.) for 3 days.

### Star Track Plasmid Vectors

Star Track plasmids (Fig. 1E) were generated as previously described [25]. Briefly, the promoter chosen to direct the expression of reporters corresponded to a 2.2 kb fragment that spans base pairs −2163 to +47 relative to the transcriptional start site of the human Gfap gene, with the normal protein-initiating ATG codon converted to TTG. This promoter was inserted in a PB-UbC-EGFP vector (Piggybac transposon plasmid encoding EGFP under the Ubiquitin C human gene promoter regulation; kindly provided by Prof. Bradley). The resulting plasmid, PB-GFAP-EGFP was subsequently used to replace the EGFP between BamHI and EcoRI with mTSapphire, mCerulean, yellow fluorescent protein (YFP), monomeric Kusabira Orange (mKO), and mCherry. To generate the nuclear forms of these markers, the human H2B histone (GenBank ID X00088.1) was amplified from genomic human DNA and upstream fused to these reporters. All plasmids were sequenced to ensure the accuracy of cloning.

### Histology and Immunohistochemistry

Seven days after lesions, animals were anesthetized with an overdose of equithesin and transcardially perfused with a 0.1% heparinized saline solution and then with 1 ml/g of ice-cold 4% paraformaldehyde. Brains were removed and sectioned in the coronal plane at 50 µm using a vibratome. Selected sections were immunolabeled with the following primary antibodies: mouse IgG anti-GFAP (1∶1000; Millipore), mouse IgG anti-βIII-Tubulin (1∶1000; Millipore); rabbit IgG anti-PDGFRalpha (1∶300; Santa Cruz Biotechnology), anti-phospho-histone H3 (pH 3) (1∶300; Upstate) and anti-Ki67 (1∶500; RabMAbs), all of them diluted in PBST 0.1 supplemented with 1% normal goat serum (NGS, Millipore). Unspecific protein binding was blocked by incubating the sections with PBS-0.1% Triton (PBST) +10% NGS for 1 h at RT and then secondary antibodies, goat anti-mouse IgG-Biotin conjugated (Molecular Probes), were incubated 2 h at RT. Labeling was visualized by incubating the sections 2 h at RT with Alexa-633 conjugated streptavidin (Molecular Probes) diluted 1∶1000 in PBST.

Fluorescent labeling was visualized using a Leica TCS-SP5 confocal. The excitation and absorption conditions for each fluorophore were (in nanometers): mT-Sapphire (Ex: 405; Ab: 525–553), mCerulean (Ex: 458; Ab: 464–481), EGFP (Ex: 488; Ab: 496–526), YFP (Ex: 514; Ab: 520–543), mKO (Ex: 514; Ab: 550–600), mCherry (Ex: 561; Ab: 601–612), and Alexa 633 (Ex: 633; Ab: 649–760).

### Cumulative Proliferation Assays

Proliferation during the 7 days post lesion was evidenced following the incorporation of the thymidine analog 5-bromo-2-deoxyuridine (BrdU) occurring during S-phase. Since usual BrdU protocols remove most of the fluorescence derived from fluorescent proteins, we performed an alternative approach. First, we employed selected electroporated animals with plasmid mixtures lacking the mKO and mCherry reporters. Starting from the next day after surgery, mice were daily given with i.p. injections of BrdU (50 mg/kg animal weight; Sigma) diluted in Tris-HCl (pH 7.6). Seven days after the lesion, animals were processed for anti-GFP immunohistochemistry as is described above (GFP antibody dilution 1∶1000; Molecular Probes) and visualized using an anti-rabbit IgG Alexa 633 conjugated antibody. This secondary antibody recognized at the same time EGFP, EYFP, mT-Sapphire and mCerulean reporters due to the structural similarities between these molecules. Images were acquired using confocal stacks showing the colocalization of the antibody staining and Star Track labeling. These same sections were processed for BrdU immunohistochemistry as follows: sections were incubated for 30 minutes at 37°C in HCl 2 N solution and finally rinsed in 0.1 M boric acid for 10 minutes at room temperature. BrdU immunostaining procedure was performed by incubation with BrdU antibody (1∶1000; Hybridoma Bank) followed by anti-mouse IgG Alexa 568 as secondary antibody. Since HCl does not affect GFP immunohistochemistry labeling, we used this labeling as bridge to compare BrdU and Star Track labeling.

### Nissl Staining

Selected sections from injured mice were processed for a Nissl staining. Coronal sections (20 µm) were stained with acid thionin (pH 4.5) for 45 seconds. Then, slices were sequentially rinsed during 1 minute on 70%, 80%, 96%, 100% ethanol and xylene, and mounted using Depex mounting media.

### Circle and Solidity Measures

Measures of circle and solidity were taken using the NIH-ImageJ software based on the cell perimeter (detailed information about the selected parameters studied available on the software website) of both protoplasmic and fibrous astrocytes (N = 15 astrocytes from 3 different animals, 5 per animal). These both parameters represented how cells are round. Circularity represents more circular cells when the value approaches 1: Circularity = 4π (area/perimeter^2^). Solidity is defined as the ratio of the convex hull area over the area: Solidity = area/convex area.

Statistical differences in circularity and solidity between protoplasmic and fibrous astrocytes were analyzed applying a t-student test using the Statistica7.0 software.

## Results

### Establishment of the Electroporation Areas before Injuries

**Figure 2:**
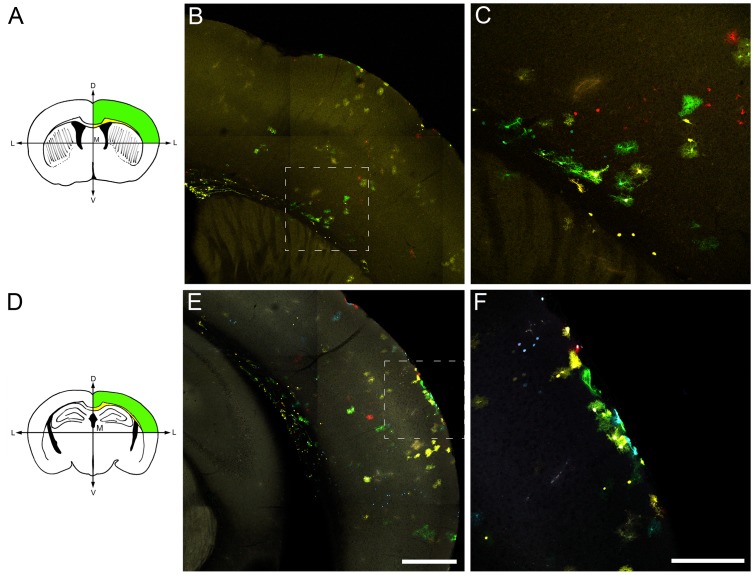
Cortical clonal dispersion in control adult mice after electroporations at E14. **A.** Representative scheme of the most anterior cortical lesion. **B.** Low magnification view of the clonal groups located at the level of the rostral coordinate chosen for the injury. The majority of clonal groups exhibit protoplasmic morphologies throughout the cortical layers. Note some clonal cells attached to the lateral ventricle with their radial processes extending from the ventricle across the corpus callosum. **C.** Detail of inset in B. **D.** Representative scheme of the posterior stereotaxic coordinate chosen for the cortical lesion. **E.** Clonal dispersion of cells with some clones attached to the pial surface with a fibroblast-morphology **F.** Detail of the inset in E. Scale bars: B,E 500 µM; C,F 200 µm.

Electroporations of the StarTrack mixture at E14 generated an inheritable mark in astrocyte lineages, visible in the most dorsal part of the cerebral cortex at adult stages. We selected two specific anterior-posterior stereotaxic coordinates that showed a maximum number of labeled astrocytes to get an optimal analysis of the lesions (Fig. 2A, D). Those astrocytes with the same code of colors were assigned to the same lineage defining astrocyte clones. The electroporations showed the same pattern of sparse clonal labeling distributed throughout the cortical thickness at both anterior (Fig. 2A–C) and posterior (Fig. 2D–F) locations. In both coordinates astrocyte clones were attached to the wall of lateral ventricles (LV), which in some cases extended radial processes from the ventricle wall to the corpus callosum (Fig. 2B, E). A characteristic feature, highlighted by the Star Track method, was the different morphologies exhibited by the astrocyte clones in all animals. Thus, some astrocytes were protoplasmic and turned into the cortex perpendicular to the ventricular surface (Fig. 2B, C) while others were attached to the pial surface with a fibroblast-morphology (Fig. 2E–F) as we previously described [25]. Since the pattern of clonal labeling was reproducible among animals, we established those anterior-posterior stereotaxic coordinates to perform anterior and posterior cortical injuries.

### Clonal Groups Revealed Different Astrocytes Morphologies in Intact Brains

**Figure 3:**
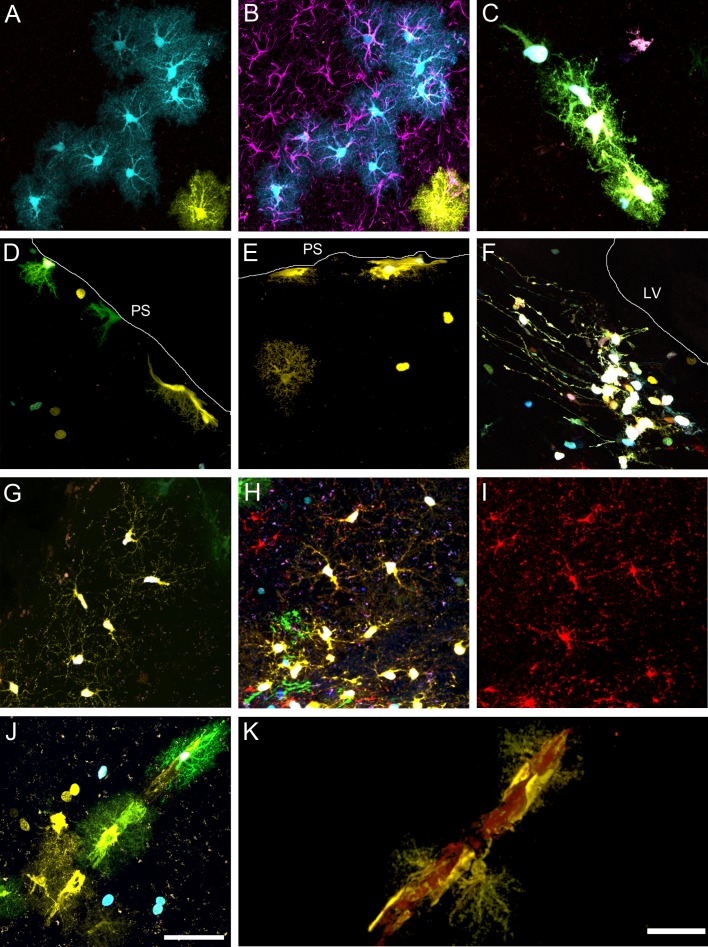
Representative astrocyte morphologies in adult cortical areas after in utero StarTrack electroporation at E14. **A.** Protoplasmic astrocytes. **B.** Immunohistochemistry against GFAP in brain sections labeled by Star Track. **C.** Immature astrocytes/progenitors, classified as a subtype of protoplasmic astrocytes. **D–E.** Pial astrocytes that extend their somas along the pial surface and emit brush cellular processes into the brain parenchyma. **F.** Radial glia in the ventricular/subventricular zone. **G–H.** Mesh-forming cells resembling NG2-glia. **I.** Immunohistochemistry against PDGFRα typically expressed by NG2 cells. **J–K.** Astrocytes radially arranged around a single blood vessel. Scale bar: 50 µm.

**Figure 4:**
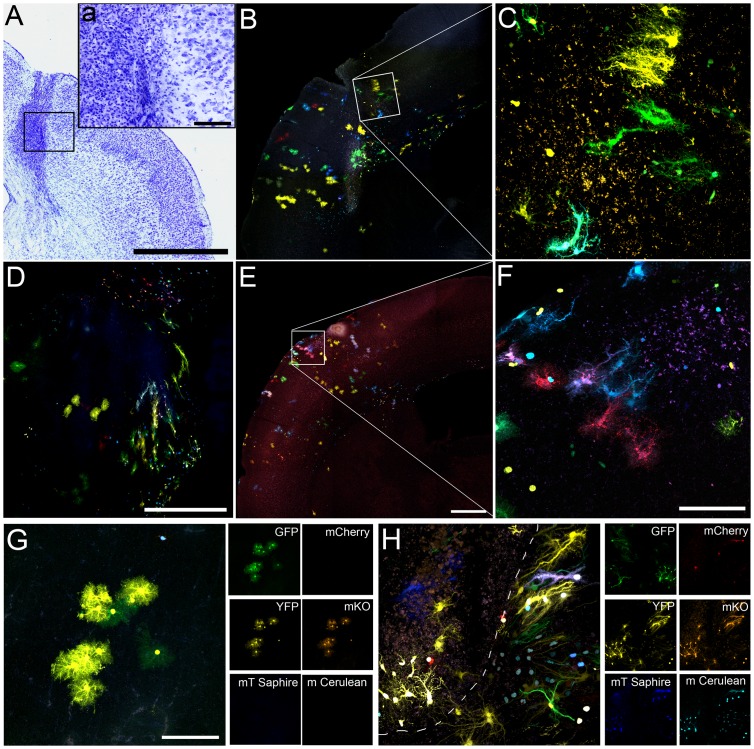
Reactive astrocyte clones in the lesion area and within the needle track. **A.** Nissl staining showing the damage after the cortical injury. Note the increment in cell density and cellular debris delimiting the needle track. **B.** Representative section of the astrocyte clones disposition around the injury. A bunch of astrocyte morphologies is observed around the injury with clones of reactive astrocytes close to the injury. **C.** Magnification of the box highlighted in B. **D.** Groups of hypertrofied astrocytes around the wounded area. **E–F.** Hypertrophied clones emitting their cell processes to the injury edge, full of cellular debri. **G.** Representative image of a typical protoplasmic clone located in intact cortical brain areas. These astrocytes are characterized by their round shapes. Small insets show merged and individual channels of small clonal groups containing cells that share the same fluorescent marks after electroporation with the Star Track mixture. **H.** Representative image of the fibrous hypertrophied astrocytes typical in reactive astrocytes after injury. Note the change from round shapes (G) by one more enlarged with thicker cellular processes (H). Scale bars: A, 1 mm; a, 100 µm; B, D, E 500 µm; C, F, G, H 100 µm.

**Figure 5:**
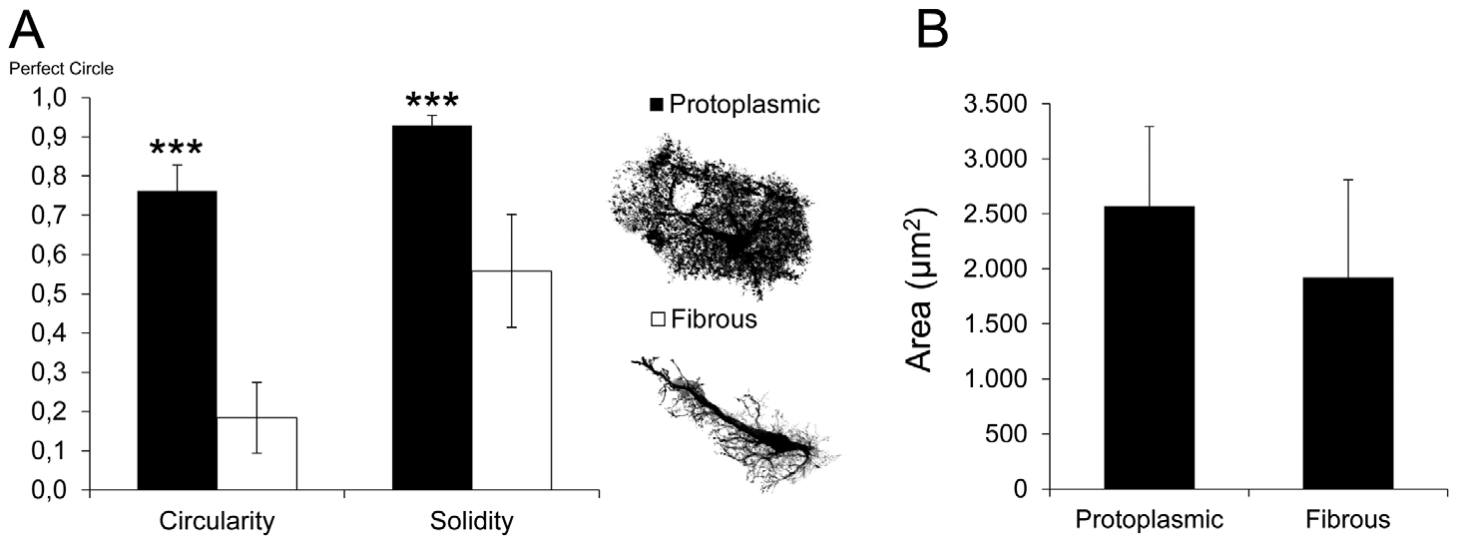
Quantitative analysis of morphological astrocyte features. **A.** The graphic represents the measures of two morphological parameters analyzed in both protoplasmic and fibrous astrocytes: circle and solidity. Both parameters are significantly lower in fibrous than in protoplasmic astrocytes indicating the lost of rounded morphologies. **B.** Estimation of astrocyte area in the different astrocyte types. None differences were evident between fibrous reactive and protoplasmic astrocytes (Graphics represent mean ± SD. Significant differences, *** are p<0.01).

The Stark Track method allowed visualizing astrocytes heterogeneity in clones based on their morphology. This method has an important advantage compared with a classical immunostaining, which is to visualize the whole cell somata, including their terminal processes, compared with the 13% of cell volume labeled after a GFAP immunostaining (Fig. 3A, B) [26]. Thus, Star Track provided an optimal resolution to identify the following type of astrocytes based on their morphology: 1) A type occupying most of the cortical extension, usually known as *protoplasmic astrocytes*, which showed spongiform shapes mostly clustered in non-overlapping processes domains (Fig. 3A, B). It was remarkable the differences in the cell body volume labeled with the Star Track method (Fig. 3A) and a classical GFAP immunohistochemistry (Fig. 3B); 2) a subtype of protoplasmic astrocytes resembling *immature astrocytes/progenitors*, which had smaller somas and shorter/thick cellular processes poorly ramified (Fig. 3C); 3) *pial astrocytes* [25], being part of the pial layer in the brain surface, which spread cellular processes into the brain parenchyma (Fig. 3D, E); 4) *radial glia* whose cell bodies were located in the ventricular/subventricular zone and extended thin radial processes along the corpus callosum (Fig. 3F); 5) *mesh-forming cells*, with small, thin and poorly branched cellular processes that spread out from a small cell soma, corresponding to NG2-glia (Fig. 3G–I). The NG2 phenotype of these cells was demonstrated by the expression of the PDGFRalpha (Fig. 3H,I); and 6) astrocytes radially arranged in a single large blood vessel (Fig. 3J, K; Movie S1).

### Hypertrophy of Astrocyte Clones in Response to the Injury

The model of fine-needle cortical injury chosen in this work affected both grey and white matter of the brain parenchyma that gave rise to a large accumulation of cellular debris and an increment in the cell density, produced by an increase in cellular migration to the injured area (Fig. 4A, a). A barrier delimiting the damaged and undamaged tissue was evident in the scared tissue (Fig. 4a). Although many type of cells react to a brain injury, this work was focused in the analysis of astrocytes labeled to identify clones. Gliosis of astrocytes clones was analyzed both in the proximity of this barrier and in the core of the injury (Fig. 4B–F). Those clones that were closer to the injury were strongly reactive showing hypertrophied cell bodies, evidenced by a strong labeling of their thick cellular processes oriented to the injury. These cells formed clones of fibrous reactive astrocytes (Fig. 4B–F, H). Remarkably, this hypertrophy was strongly asymmetric, with long and thick processes pointing out to the injury core and few and short processes in the opposite side (Fig. 4C, F, H). In an overview, the wounded region resembled an attractor for cells and processes (Fig. 4D). All fibrous-reactive clones were characterized for changing their typical round shapes by one more enlarged with thicker cellular processes (Fig. 4G–H), as shown after the analysis of the circularity and solidity morphological parameters (Fig. 5A). This change in morphology did not affect to the area of cell bodies (Fig. 5B).

### Heterogeneity in the Clonal Response to the Cortical Lesion

**Figure 6:**
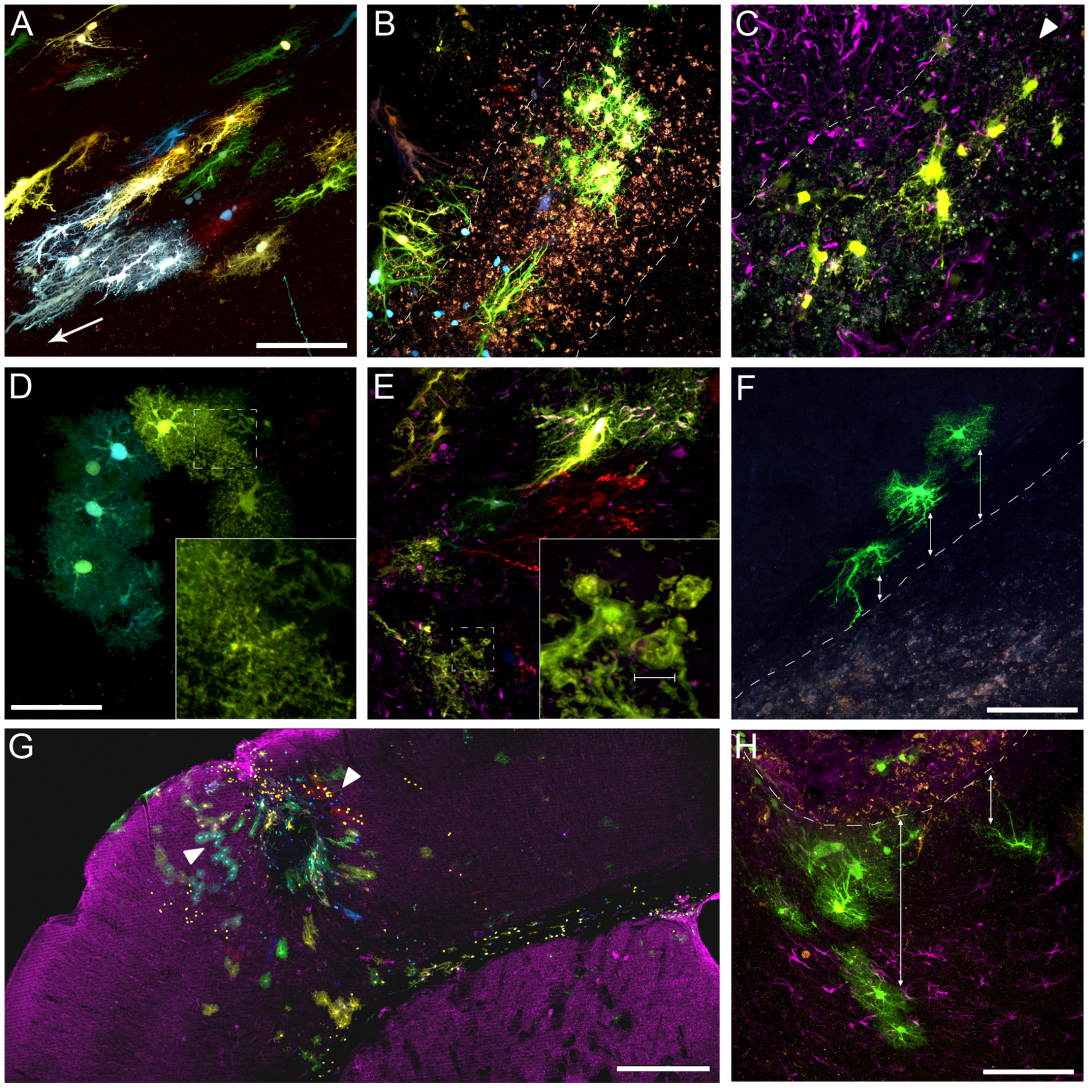
Heterogeneity in astrocyte clones depending on their position with respect to the injury. **A.** Clones located close to the injury, adopted the classic hypertrophied morphology with enlarged cytoplasm and thick cellular processes oriented to the injury border **B–C.** Reactive clones sited into the injury show smaller sizes and acquire a mesh-like morphology. GFAP immunohistochemistry is represented in magenta. **D–E.** Reactive clones show cytoplasmic protrusions in the terminal cell processes, with a constant diameter size of 5 µm. **F.** Representative clone shows a change in cell morphology from protoplasmic to a fibrous/hypertrophied as their cells approach to the needle track. **G.** Low magnification view of different reactive clonal groups around the lesion area. It is remarkable the different astrocyte responses in relation with the distance to the injury. Some astrocyte clones are entirely protoplasmic (arrowheads) while others are highly hypertrophied. The magenta represents an immunohistochemistry against B-III-Tubulin neuronal marker. **H.** Clonal group of astrocytes changing their morphologies as they approach to the injury in a section processed for GFAP immunohistochemistry (magenta). Scale bars: A 100 µm; B, C, D, E 50 µm; F 100 µm; G 500 µm;

Most astrocyte clones exhibited heterogeneous identities around the lesion area. Thus, those clones located close to the injury exhibited hypertrophic morphologies with enlarged cytoplasm and thick cellular processes oriented to the injury border (Fig. 6A, arrow). However, clones situated within the needle track were smaller in size with short/thin cellular processes and acquired a mesh-like structure (Fig. 6B, C). Unlike the protoplasmic astrocytes, reactive clones showed cytoplasmic protrusions in the terminal cell processes, with a constant diameter size of 5 µm (Fig. 6D, E).

Since different type of astrocytes arise from specialized progenitor cells [25], we expected that siblings astrocytes responded equally to the brain damage. However, a concise examination of the clones next to the injury revealed different clonal responses. Thus, some reactive clones acquired hypertrophic morphologies in relation to distance to the injury (Fig. 6F–H, arrowheads), while others did not modify their morphologies, even at the same distance from the injury (Fig. 6G, arrowheads). Taken together, our data suggests that astrocytes belonging to a same clone were developmentally determined to respond differently to an injury.

### Clonal Proliferation in Response to the Injury

**Figure 7:**
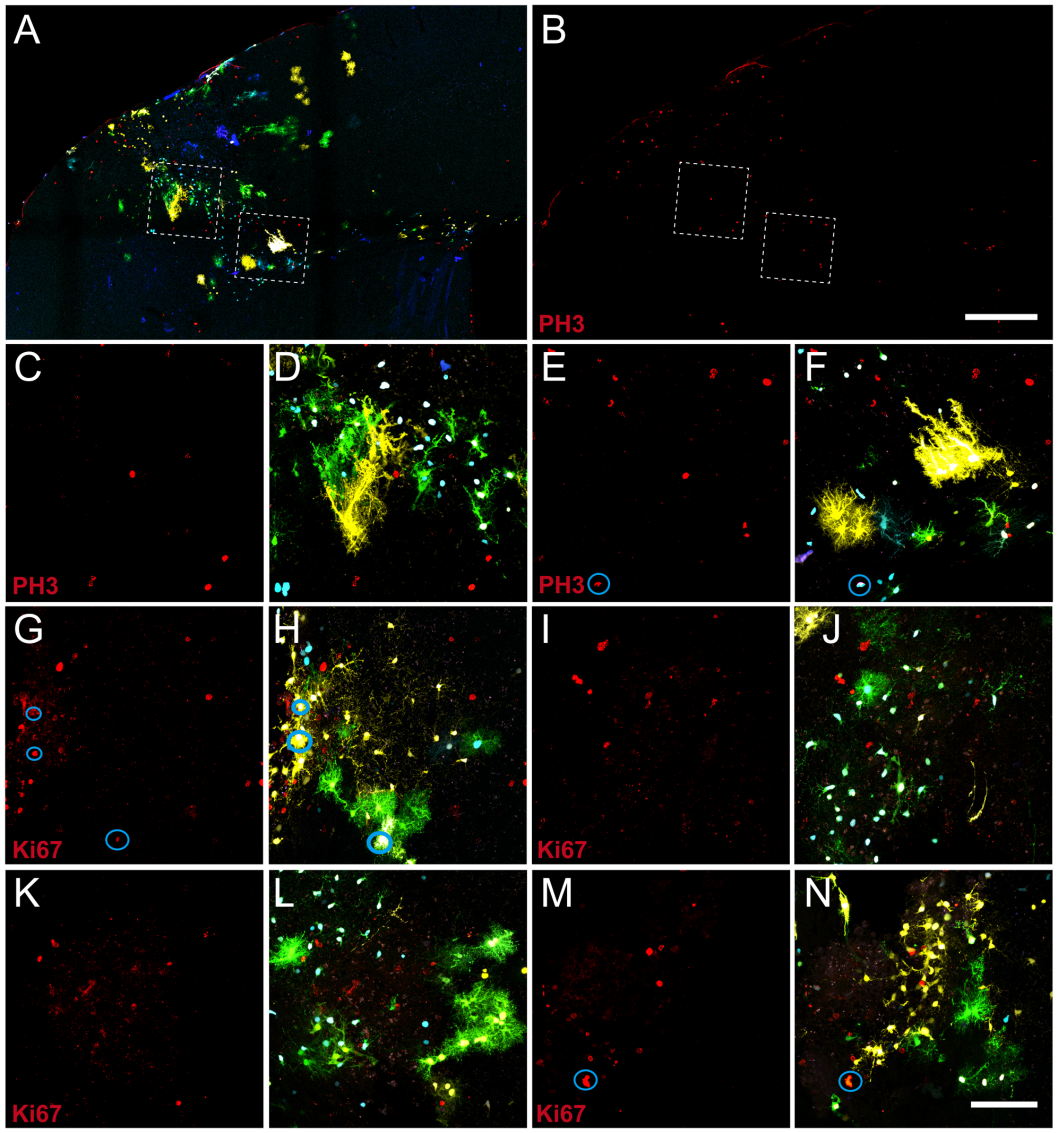
Cell proliferation detected by iMmunohistochemistry against pH 3 and Ki67 proliferative markers at seven days post injury. **A.** Low magnification of the injured hemisphere showing the merge of the Star Track staining with the labeling against the proliferative marker pH**B.** pH 3 labeling of the image in (A). **C–F.** High magnifications of the regions highlighted in A–B. **C–E** PH 3 labeling, **D–F** pH 3 channel merged with the Star Track labeling. **G, I, K, M.** Labeling of the Ki67 inmunohistochemistry. **H, J, L, N.** Merging of the Star Track and Ki67 labeling. Blue circles indicate those cells labeled by the Star Track co-expressing the proliferation markers pH 3 and Ki67. Blue circles indicate those Star Track labeled cells co-expressing proliferation markers. Scale: A–B 400 µm; C–N 100 µm.

Open injuries correlated with a significant increase of the cellular proliferation inside and surrounding the damaged area. This proliferation characterizes the reactive astrocyte response [5]–[8]. To elucidate the relationship between clonal organization and proliferation, we performed immunohistochemistries for the proliferation markers pH 3 and Ki67 at seven days post injury (Fig. 7A–N). As expected, the presence of dividing cells was critically related to the lesion (Fig. 7A–B). In the injured area, the distribution of dividing cells was fairly homogeneous. However, the number of dividing cells belonging to labeled astrocyte clones was very low (Fig. 7C–N). This suggests that the proliferative peak after 7 days post-injury was related to other cellular types located within the injured area, while the proliferation of astrocytes was earlier in the time. Another possibility is that the number of proliferating cells in each instantaneous moment is too reduced to detect clonal proliferation patterns. The expression of pH 3 was only observed in one case, in two cells of the same clone (Fig. 7G–H). Moreover, this proliferation was also very low in NG2 cells, where we did not observe any labeled cell.

**Figure 8:**
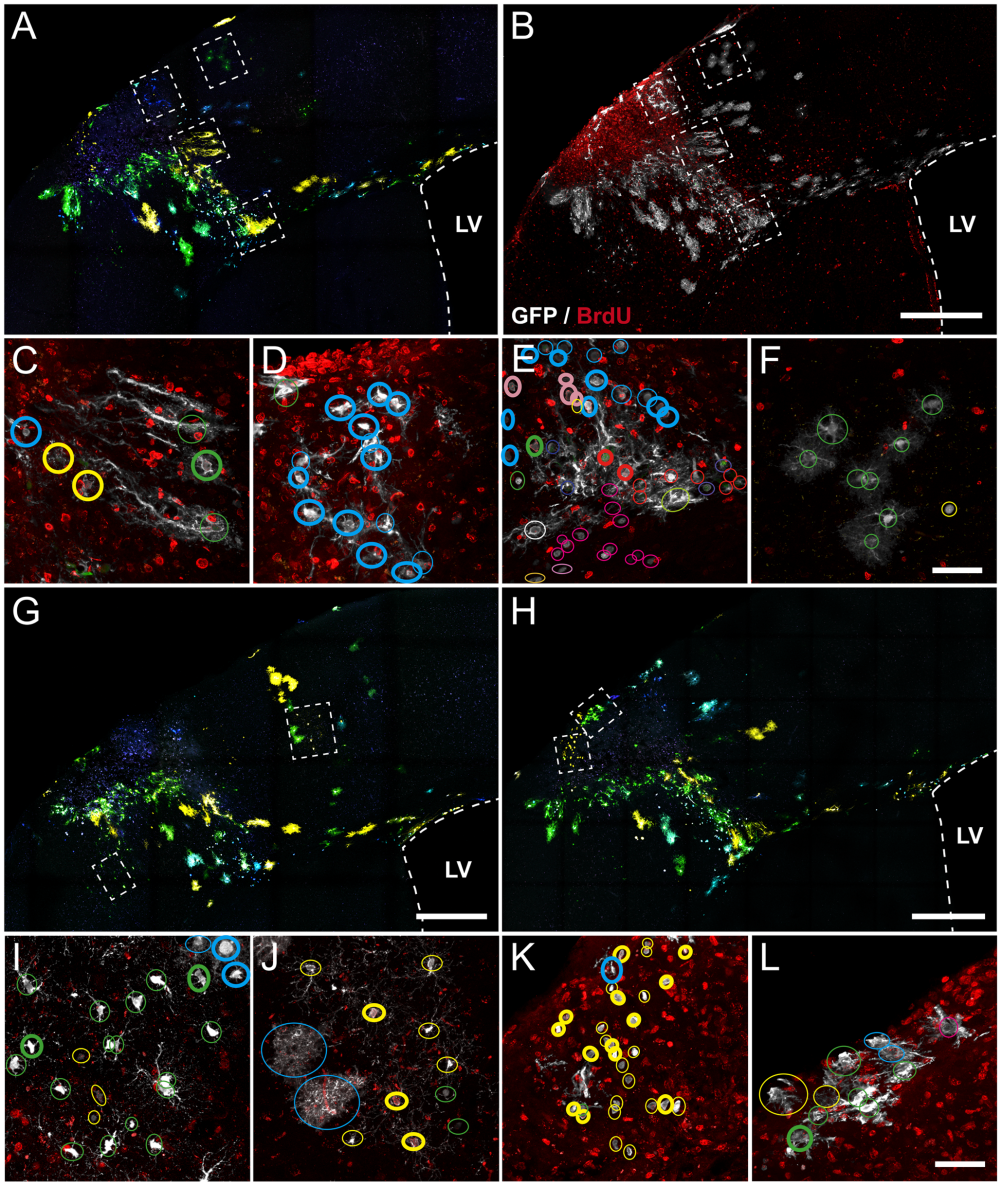
Cumulative proliferation assay based on the BrdU incorporation during seven days after injury. **A.**Low magnification image showing the Star Track clonal labeling. **B.** Same image showing immunohistochemistry against GFP (grey) and BrdU (red). C–F. High magnification of areas highlighted in insets of A–B. **G–L.** Additional sections labeled with Star Track and GFP/BrdU immunohistochemistry. **G–H.** Images at low magnification showing the Star Track labeling. **I–L.** High magnification images of regions highlighted in insets. For each image, cells that are part of the same clone are encircled. Thick circles represent those cells that incorporated BrdU. Scale: A–B, G–H 400 µm; C–F, I–L 50 µm.

Since the proliferation snapshot at seven days post injury was insufficient to discern clonal proliferation patterns, we labeled the total number of dividing cells during this time-frame by a modified BrdU assay. Similar to pH 3 and Ki67, BrdU revealed a significant accumulation of dividing cells inside and surrounding the lesion area (Fig. 8A–L). However, the number of dividing cells labeled by this approach was much higher, and were distributed both inside and surrounding the lesion. In those areas close to the injury were observed a high proportion of Star Track labeled cells containing this thymidine analog (Fig. 8C–E, K), in contrast to those cells located far from the injury (Fig. 8F). BrdU assay showed an apparent clonal response in terms of cell proliferation. As evidenced in Fig. 8C–E, most of cells being part of some clones (yellow, blue and pink clones) were de novo originated, in contrast to other clones in which the majority of their cells were present before the lesion (magenta and red clones). This response was also elicited in NG2 clones, in which the proportion of dividing cells per clone was even lower in some clones closer to the lesion, in contrast to other clones with higher proliferation in distant regions (Fig. 8I–J). In pial clones, the number of proliferating cells is very low compared to their distance to the lesion (Fig. 8L).

The lack of BrdU in some clonal cells indicates that the astrocyte proliferative response is mainly due to resident clones, albeit the clonal and proliferation pattern also suggest a cellular contribution from the subventricular zone. Moreover, the proliferation rate rapidly decays with the distance, evidencing the proliferation signals derived from the lesion acting at local distances.

## Discussion

This work addresses important aspects on the relationship between astrocyte lineage and their response to injury. Our results demonstrate that groups of astrocyte clones respond differentially to cortical injury. Whereas in some clones all sibling cells showed the typical hypertrophy of reactive astrocytes, other clones, located at comparable distances, did not display altered cell morphologies. Moreover, in other cases only some cells of the same clone showed hypertrophied cytoplasm, closely related to the distance to injury (Fig. 6F). These data indicate that intrinsic factors determined during the embryonic development affect the astrocyte clones depending on their lineages. On the other hand, some astrocytes exhibited a clonal response in terms of proliferation. Whereas most of the cells forming some clones were generated de novo, other clones at similar distances showed limited cell proliferation. In this case, the proliferative response indicates that there are both intrinsic and extrinsic factors secreted in the injured environment, which affect directly to the ability of cell proliferation. This variability in the clonal response to injury clearly indicates that the heterogeneity of reactive astrocytes could be determined both at developmental stages, depending on their lineage, and because of different signals present in the environment after the injury.

Heterogeneity in reactive astrocytes has been described over the past twenty years and many factors have been shown mediating the process of reactive gliosis [27], [28]. However, despite the growing interest in the study of astrocyte heterogeneity, many issues remain unresolved. At this respect, we recently developed a new method for cell clonal analysis named *Star Track* that allows to study astrocyte lineages by the expression of different fluorescent proteins under the regulation of the GFAP promoter [25]. In that work we reported that different type of astrocytes are determined early in the development from a single cell, giving rise to a cell clone. Given the existence of astrocyte heterogeneity after injury [20]–[22], we applied the *Star Track* method to analyze the clonal response to cortical lesions.

Our data show that the response of astrocyte clones to a cortical injury after one week, coincident with both the peak of cell proliferation and the upregulation of GFAP and vimentin [29], [30], is heterogeneous and in some cases dependent on the distance to the injury. Thus, some clones responded with all of their cells hypertrophied while others, at similar distances to the lesion, exhibited a non-reactive phenotype. Given that, *a priori*, cells belonging to the same clone were exposed to the same molecular environment, these findings point to the existence of intrinsic developmental mechanisms codified in their lineage that are responsible for this heterogeneous response to injury.

However, while most clones responded homogeneously as a whole, some clones showed some of their cells gradually hypertrophied in relation to the distance to injury. Since astrocytes are connected via gap-junctions forming networks in response to neuronal activity [31], [32], it would be expected a similar response of sibling astrocytes. However, the fact that some sibling astrocytes in the same clone responded differently in relation to the injury suggested that the reactive response was independent to the coupling of astrocytes and, therefore, related to factors secreted in the environment (extrinsic responses). In this regard, some experiments suggested certain degree of uncoupling since some astrocyte K^+^ currents change in relation to the distance to injury [33]. Although further experimentation will be required to establish if a brain injury induces loss of astrocyte coupling, this could be related to the heterogeneity in the clonal response.

The intrinsic/extrinsic responses of astrocytes to an injury were also evident after the study of cell proliferation. Thus, reactive astrocytes increase in number after an injury, but it is still controversial the source of these astrocytes and their relation with the lineage. We analyzed the cell proliferation within the clones in the injured area, showing that the majority of both astrocytes and NG2 clones exhibited a proliferative clonal response. A detailed analysis of the proliferation markers indicated that whereas some clones exhibited a poor proliferative response other clones, located at similar distance to the lesion, displayed the majority of their cells as new-generated. This data suggested that intrinsic factors were acting when these cells decided to proliferate.

Even though these results suggest a heterogeneous clonal response codified in their lineage, the intrinsic response to the injury, based in clones consisting completely by new generated cells, could be explained by an alternative hypothesis. Progenitors in the SVZ can proliferate in response to a lesion, giving rise to glial progenitors that migrate throughout the corpus callosum to cortical areas [34]. Since they are produced from SVZ stem cells, these migratory progenitors and their whole progeny should contain BrdU. However, in our BrdU experiments we could not find any clone completely comprised by BrdU positive cells, questioning the relevance of this cellular immigration and the existence of extrinsic factors affecting cell proliferation. Thus, we concluded that this heterogeneous clonal response should be determined during the development of these clones. Similarly, these findings also showed a close correspondence between proliferation and distance to the lesion. In concordance with previous estimations [23], [35], [36], proliferation of resident astrocytes is not noteworthy and it rapidly decays with the distance. Only those clones inside or in the border of the lesion exhibited a significant proliferative response, suggesting a releasing of diffusible molecules from the injured area acting at short distances. This new rationale combining both BrdU and Star Track will be tremendously helpful in future studies to discern both types of cellular contributions.

As long as Star Track outlines the morphology of individual cells, it allows understanding many crucial points of the glial response to lesion. For example, it is showed the link between distance to lesion and the hypertrophy of cellular processes. Indeed, the strong asymmetry of hypertrophied processes suggested the occurrence of chemotactic mechanisms determining this reaction. Several diffusible molecules could be responsible of this process, including factors such as transforming growth factor-beta 1 (TGF-beta 1), basic fibroblast growth factor (bFGF), epidermal growth factor (EGF), transforming growth factor-alpha (TGF-alpha), and heparin-binding epidermal growth factor [37].

Star Track provides *in vivo* evidences of how development leaves a fingerprint of heterogeneity in the capacity of astrocytes responding to an injury. Taken together, these findings indicate the existence of intrinsic factors, determined during the embryonic development and related to the cell lineage, which affect the astrocyte response to a brain injury. These factors are not exclusive from others secreted in the environment that also affect differentially to astrocyte clones. This implies that there is a high heterogeneity in the astrocyte responses to injury suggesting the importance of designing selective strategies to promote the CNS repair.

## Supporting Information

Movie S1 **Three-dimensional reconstruction of a Star Track astrocyte clone covering a single blood vessel.**
(AVI)
